# Iron Metabolism Disturbance in a French Cohort of ALS Patients

**DOI:** 10.1155/2014/485723

**Published:** 2014-07-02

**Authors:** Charlotte Veyrat-Durebex, Philippe Corcia, Aleksandra Mucha, Simon Benzimra, Cindy Mallet, Chantal Gendrot, Caroline Moreau, David Devos, Eric Piver, Jean-Christophe Pagès, François Maillot, Christian R. Andres, Patrick Vourc'h, Hélène Blasco

**Affiliations:** ^1^Unité Mixte de Recherche U930, Institut National de la Santé et de la Recherche Médicale, Université François-Rabelais, Equipe “Neurogénétique et Neurométabolomique”, 10 boulevard Tonnellé, 37032 Tours, France; ^2^Laboratoire de Biochimie et de Biologie Moléculaire, Hôpital Bretonneau, Centre Hospitalier Régional Universitaire de Tours, 37044 Tours, France; ^3^Centre SLA, Service de Neurologie, Centre Hospitalier Régional Universitaire de Tours, 37044 Tours, France; ^4^Service de Neurologie, Centre Hospitalier Régional Universitaire de Lille, 59037 Lille, France; ^5^Laboratoire de Biochimie, Hôpital Trousseau, Centre Hospitalier Régional Universitaire de Tours, 37044 Tours, France; ^6^Service de Médecine Interne, Centre Hospitalier Régional Universitaire de Tours, 37044 Tours, France

## Abstract

*Objective.* The aim of this study was to assess iron status in a cohort of amyotrophic lateral sclerosis (ALS) patients compared to controls in order to evaluate these parameters as a risk factor or a modifying factor of ALS. *Methods.* We collected serum iron, ferritin, transferrin, total iron-binding capacity, and transferrin saturation coefficient (TSC) from 104 ALS patients at the time of diagnosis and from 145 controls. We reported phenotypic characteristics and evolution parameters such as ALSFRS-R and forced vital capacity at diagnosis and after one year of follow-up. In a first step we compared iron status between ALS patients and controls, and then we evaluated the relation between iron status and disease evolution of ALS patients using univariate and multivariate analysis. *Results.* We observed increased concentrations of serum iron (*P* = 0.002) and ferritin (*P* < 0.0001) and increased TSC (*P* = 0.017) in ALS patients. We also showed an association between markers of iron status and high body weight loss in ALS patients. The multivariate analysis of survival highlighted a significant relation between ferritin level and disease duration (*P* = 0.038). *Conclusion.* This is the first study showing a higher concentration of serum iron in ALS patients, strengthening the involvement of a deregulation of iron metabolism in ALS.

## 1. Introduction

Amyotrophic lateral sclerosis (ALS) is a fatal neurodegenerative condition characterized by degeneration of both lower and upper motor neurons. Genetic factors, including mutations of the* C9orf72*,* SOD1* (superoxide dismutase 1),* TARDPB,* or* FUS* (fused in sarcoma) genes, may explain almost 50% of familial cases (FALS) [1]. The etiology of this adult-onset motor neuron disease remains unknown for many sporadic cases (SALS). Other factors such as oxidative stress, glutamate-mediated excitotoxicity, hypoxia, mitochondrial dysfunction, cytoskeletal abnormalities, and protein aggregation might have a causative role in ALS [2].

Iron is essential to life and plays a role in many neuronal functions; various neurodegenerative diseases such as Parkinson and Alzheimer diseases are associated with iron metabolism deregulation [3, 4]. Signs of iron accumulation and high levels of iron import have been observed in the central nervous system of ALS patients [5–7] and in a mouse model of ALS [8]. Several clinical reports showed higher serum ferritin and lower serum transferrin levels in ALS patients [9–12]. Recently, Nadjar et al. suggested that high ferritin levels were associated with poor survival in ALS men [11]. Also, transferrin has been found in Bunina bodies within motor neurons from ALS patients [13]. Two genes involved in iron metabolism have been studied in ALS patients:* HFE* mutated in cases of hereditary hemochromatosis and* SLC11A2* encoding the divalent metal transport 1 (DMT1) protein. Accordingly, several studies tend to link a rare allele of* HFE* (mutation H63D) and ALS [14–16]. However, this association is not replicated in all studies [17]. Although no association could be established between* SLC11A2* rs407135 variant and SALS, this polymorphism is associated with a rapid disease evolution among ALS patients with lower limb onset [18]. Altogether, these various observations are consistent with iron metabolism disturbances in ALS. However, only partial analyses of iron metabolism have been reported in ALS patients so far and no systematic assessment of its role in disease evolution has been conducted.

We reported here the comparison of the iron status at the time of diagnosis for ALS patients with that of a series of paired controls. We also included most of relevant parameters directly or indirectly linked to iron metabolism. Finally, we evaluated the relationship between these biological parameters of iron metabolism and both the phenotype and the survival of ALS patients.

## 2. Material and Methods

### 2.1. Patients

We analyzed data from 104 SALS patients and 145 controls (recruited from May 2008 to June 2013). Blood samples were obtained at the time of diagnosis in the French ALS center of Tours. The diagnosis of ALS was made according to diagnostic criteria for definite or probable ALS based on the El Escorial World Federation diagnostic criteria [19]. For each patient, clinical data including information on diagnosis, gender, age, site of onset, age at onset, and disease duration were obtained. The site of onset was defined as either bulbar or limb onset. Bulbar onset was defined as symptoms first occurring at the bulbar level with dysphagia, dysphonia, or dysarthria. Limb onset was defined as symptoms first occurring in the limbs. The age at onset was defined as the time at which motor weakness was first noted by the patient. The disease duration of ALS was defined as the time since onset (first symptoms) or between onset and death or tracheostomy. We collected ALSFRS-R (ALS functional rating scale-revised) and forced vital capacity at diagnosis and after one year of follow-up. Progression rate (ΔFS) was calculated as described by Kimura et al. [20], as ΔFS = (48-ALSFRS-R at diagnosis)/duration from onset to diagnosis (month). We also took into account the loss of body weight at diagnosis (difference between reference weight reported by the patient and weight at diagnosis). We collected genotype status for* HFE* gene polymorphisms C282Y and H63D. Control group included hospitalized individuals with no neurological disorders, no history of iron metabolism disorders, and no inflammatory disease, coming from the same geographical origin, and matched with ALS patients for age and sex.

### 2.2. Biochemical Evaluations

The iron status of patients and controls was evaluated by measuring serum iron, serum transferrin, total iron-binding capacity (TIBC), transferrin saturation coefficient (TSC), and serum ferritin. Plasma creatinine and C-reactive protein were also assayed. In order to verify that differences in iron metabolism were not due to defective renal elimination, creatinine value was used in the MDRD (modified diet in renal disease) equation to estimate the glomerular filtration rate (eGFR). C-reactive protein was assayed to define the inflammatory status of patients at the time of biochemical exploration. Iron concentrations were determined by a colorimetric method with an AU2700 apparatus (accuracy with relative standard deviation RSD = 2.1%) and ferritin concentrations by a chemiluminescence method with an Access 2 (RSD = 6.3%) (Beckman Coulter). Transferrin was determined by immunonephelometry using a BN ProSpec system (RSD = 4.8%) (Siemens Healthcare Diagnostics). The TIBC (*μ*mol/L) was calculated using the following formula: serum transferrin (g/L) × 25. The TSC was calculated using the equation (serum iron (*μ*mol/L)/TIBC × 100). Signed consent was not required for these biochemical tests because they were part of usual clinical care as approved by the ethics committee of Tours Hospital (CPP, for Comité de Protection des Personnes). The ethic committee of Tours Hospital approved this study.

### 2.3. Statistical Analysis

The Chi² test was used to compare sex ratios between ALS and controls groups and the Wilcoxon nonparametric test to compare mean age and biochemical measure values.

We evaluated the relation between each biological parameter and clinical data using Wilcoxon nonparametric test (for gender and site of onset phenotypic data) and Pearson's correlation coefficient (for other clinical data). We also performed a survival analysis to evaluate the influence of biochemical parameters on the disease duration. Multivariate Cox proportional hazard models were used to explore factors associated with survival, including demographic and clinical data. All covariates characterized by a *P* < 0.2 when evaluated in univariate survival analysis were included in the Cox model. Differences were considered significant at *P* < 0.05.

Statistical analysis was performed with JMP statistical software version 7.0.2 (SAS Institute, Cary, North Carolina).

## 3. Results

### 3.1. Subjects

The characteristics of ALS patients and controls are reported in Table 1. The average age at diagnosis for the ALS patients was 67.6 years (±9.6) and the sex ratio (male/female) of 1.08 was not significantly different from that of the controls. CRP concentrations did not differ significantly between the two groups (*P* = 0.956). The plasma creatinine concentration was lower in ALS patients than in controls (*P* < 0.0001); thus, the estimated glomerular filtration rate (eGFR) calculated with the MDRD equation was higher (*P* < 0.0001). Comparisons of CRP, creatinine, and MDRD values between the ALS and control groups did not reveal any sex-effect within either group (data not shown). At diagnosis, 44% of ALS patients had lost more than 5% of their body weight. No patient underwent a tracheostomy at the terminal stage of the disease.

The averages of diagnostic delay and progression rate (ΔFS) were, respectively, 14.9 months (±18) and 1.2 (±1.1). ALSFRS-R and forced vital capacity at diagnosis and after one year of follow-up are shown in Table 2.

The genotype frequencies of* HFE* gene polymorphisms C282Y and H63D were consistent with those previously reported for control subjects [21] (*P* = 0.291 for C282Y and 0.772 for H63D).

### 3.2. Comparison of Iron Metabolism between ALS Patients and Controls

Serum iron and ferritin values were available for 104 ALS patients and 145 controls; the other markers of iron status were available for only 87 ALS patients and 135 controls. Iron (*P* = 0.002) and ferritin (*P* < 0.0001) concentrations, as well as TSC (*P* = 0.017), were all higher in ALS patients as compared to controls (Table 3). Analysis of these values according to gender revealed the same difference for serum iron and ferritin for the two sexes; however, TSC significantly differed only for women (*P* = 0.041) (Table 3).

There was no correlation between serum iron and ferritin both in the ALS and the control group (data not shown).

### 3.3. Relationship between Parameters of Iron Metabolism and ALS Clinical Data

Iron status within the ALS group was analyzed by gender and age. Serum ferritin concentrations were higher in men than in women (*P* = 0.008). No correlation was found between iron status and age. There was no significant association between the biochemical markers of iron metabolism and the site of onset (data not shown). No correlation was observed between iron status parameters and diagnostic delay (data not shown).

There were, however, significant positive correlations between serum iron and both ALSFRS-R at diagnosis (*n* = 98, adjusted *R*-squared 0.035, *P* = 0.036) and forced vital capacity at diagnosis (*n* = 73, adjusted *R*-squared 0.064, *P* = 0.018).

Low serum transferrin concentrations and TIBC were associated with a higher body weight loss at diagnosis in ALS patients (*n* = 72; adjusted *R*-squared 0.1245, *P* = 0.001 for correlation between transferrin and loss of weight and adjusted *R*-squared 0.1292, *P* = 0.001 for correlation between TIBC and loss of weight). High serum ferritin tended to be associated with a higher body weight loss at diagnosis (*P* = 0.06).

### 3.4. Relationship between Parameters of Iron Metabolism and Disease Evolution

No correlation was observed between iron status parameters and ΔFS (data not shown). We have not observed any correlation between ALSFRS-R or FVC decline in the first year and iron metabolism parameters (data not shown).

The median disease duration was 41.2 months. The univariate analysis showed that creatinine concentration was associated with disease duration (*P* = 0.026), as well as ΔFS (*P* < 0.0001), body weight loss at diagnosis (*P* = 0.017), and ALSFRS-R decline during the first year after diagnosis (*P* = 0.017). Moreover, FVC decline and serum ferritin concentration had *P* value inferior to 0.2 (0.07 and 0.11, resp.). After inclusion of these parameters in the multivariate Cox model, we observed shorter disease duration in patients with faster progression (ΔFS *P* = 0.026, HR = 3.11; ALSFRS-R decline *P* = 0.012, HR = 1.08), with a higher body weight loss at diagnosis (*P* = 0.0007, HR = 1.38), and with higher serum ferritin concentrations (*P* = 0.038, HR = 0.99).

## 4. Discussion

This study is the first to assess both diverse criteria of ALS disease evolution and several biological markers allowing appropriate interpretation of any disturbances of iron metabolism. In particular, we report that serum iron and ferritin concentrations, and TSC, were all higher in our French ALS cohort than in controls. Surprisingly, higher serum iron levels were associated with better ALSFRS-R and better forced vital capacity at diagnosis, suggesting the absence of deleterious effect at this stage of the disease. Lower serum transferrin and TIBC in ALS patients were associated with greater body weight loss at diagnosis, a poor prognostic factor. We also observed that serum ferritin concentration was associated with disease duration as suggested by Nadjar et al. [11].

Our sample of ALS patients was representative of the general ALS population, according to their age of onset and the proportion of limb onset [22, 23]. As expected, body weight loss at diagnosis, progression rate, and ALSFRS-R slope during the first year following diagnosis were associated with disease duration [24]. Our analysis confirmed previous reports showing that* HFE* polymorphisms were not associated with ALS [17].

Serum creatinine concentrations were lower in ALS patients than in controls. Creatinine is a metabolite of muscle creatine. Muscle weakness and wasting are dominant symptoms of ALS and cause respiratory failure leading to death [25, 26]; this may explain the low serum creatinine concentrations observed in these patients. The association of low plasma creatinine levels and disease duration confirms reports establishing a link between creatinine drop and the annual decline of ALSFRS-R [12, 27]. This phenomenon has a dual origin, the undernourishment of ALS patients and progressive muscular weakness, both associated with poor prognosis [24].

The higher serum iron concentration of ALS patients is consistent with reports of iron accumulation in spinal cords of motor neuron disease patients [28] and in microglial cells of the motor cortex in ALS patients [29]. Microglia regulates both oxidative stress and inflammation within the nervous system and also iron homeostasis. Deregulation of iron metabolism has been observed in neurodegenerative diseases, such as Parkinson and Alzheimer diseases, and also ALS [30]. Iron may contribute to the formation of reactive oxygen species (ROS) by the Fenton reaction, which promotes oxidative stress, one of the mechanisms involved in ALS [2, 31]. Hence, labile iron may be deleterious to DNA, proteins, and lipids [32]. Here, we report the first observational study showing higher serum iron concentrations in ALS patients. The power of this statistical analysis is more than 88%, thus being promising. A recent study showed that, in culture, neurons overexpressing the SOD1 mutant G93A had higher iron levels as compared to control cells, a feature associated with ROS generation [33]. This is consistent with findings for the SOD1G93A mouse model showing that these mice have high iron levels and that iron chelators extend their life span [34]. Moreover, nonapoptotic cell death, called ferroptosis, involves iron-dependent oxidation [35]. We could hypothesize that there is a release of free ferrous iron secondarily to oxidative stress. This would enable an oxidative phosphorylation but a toxic effect is produced by the ROS generation; then, a protection mechanism transforms free ferrous iron in ferric iron in order to store it by fixation to the ferritin. Interestingly, this study suggested that high serum iron concentration may be a marker of better condition at diagnosis but is probably not a marker of disease progression. A protective effect of serum iron concentration was shown in Parkinson disease but the mechanism remains unclear as well as the mechanism regulating the relationship between serum and brain iron concentrations [36]. A recent study highlighted the key role of blood-brain barrier astrocytes in the iron efflux in the central nervous system [37]. Glial cells are known to play a central role in motor neurons degeneration in ALS [2]. Thus, we could hypothesize that a dysregulation of astrocytes function in ALS pathology could result in a higher efflux of iron in the brain or that the breakdown of the blood-brain and blood-spinal cord barriers could be responsible for iron accumulation in central nervous system [38]. We could therefore suggest that a higher serum iron at diagnosis could reflect a better astrocytes function or a better blood-brain and blood-spinal cord barriers integrity.

High serum ferritin concentrations have previously been reported in ALS patients [9–11, 39]. Since we noticed no difference in CRP concentrations between ALS patients and controls, the higher serum ferritin concentration observed was not due to an inflammatory condition. Ferritin, a major iron storage protein, may have an antioxidative role in cells by binding free iron [40]. Goodall et al. suggested that high serum ferritin concentrations in ALS patients could be involved in the storage of the excess of free iron generated by muscle degradation releasing heme-containing myoglobin [9]. Hyperferritinemia is not a specific marker of iron accumulation and could be found during chronic inflammation or renal failure. Although it remains difficult to explore renal failure from creatinine data in ALS patients, an inflammatory status has been dismissed for these patients. High serum ferritin concentration in the ALS population was observed in a context of high TSC, which is consistent with previous findings [11]. We also confirmed that high serum ferritin concentration was associated with reduced disease duration [11], but this association highlighted by the Cox model should be interpreted with caution. According to the values of hazard ratios (HR), we could consider that the predictive role of ferritin and ALSFRS-R does not seem robust.

Our observations showed an association between markers of iron status (low serum transferrin and TIBC) and higher body weight loss at diagnosis in ALS patients. Low serum transferrin together with high iron concentrations has been described in malnourished patients [41, 42]; it is thus plausible that iron metabolism disturbance could be linked to the nutritional status of ALS patients, which may already be disturbed at diagnosis. Although body weight loss at diagnosis has been widely described as a poor prognostic factor in ALS [43], as shown in the present study, we did not observe the same relation with several markers of iron metabolism (transferrin and TIBC) linked to body weight loss at diagnosis. We noted that ferritin had a trend to be associated with the body weight loss at diagnosis and had been associated with disease duration. This association between serum ferritin and disease duration is consistent with a previous study by Nadjar et al. [11]. Unlike Nadjar et al. we did not observe lower serum transferrin in ALS patients. This could be explained by the smaller number of subjects and thus lower statistical power in our study. Alternatively, ALS population studied by Nadjar et al. could be biased, since it appeared to display substantial body weight loss associated with decreased serum transferrin concentration. According to the crucial role of hepcidin in iron metabolism, it would be informative to evaluate hepcidin status in ALS patients in order to assess its involvement in the identified iron metabolism alterations. We have to plan the evaluation of this parameter in the future studies on iron metabolism in ALS.

Finally, we could guess that the accumulation of iron observed by previous reports in the nervous system of ALS patients is linked to serum iron and ferritin concentrations at time of diagnosis, as shown in our study. Further investigations are needed to elucidate the role of iron metabolism in ALS pathophysiology. The perturbations of iron metabolism observed in our ALS population that strengthen the previous studies may be suggestive of a new pathophysiological pathway for ALS disease or reminiscent of the oxidative stress hypothesis.

## Figures and Tables

**Table 1:** Characteristics of ALS patients and controls.

	ALS (*n* = 104)	Controls (*n* = 145)	*P* value
Mean age (years ± SD)	67.6 ± 9.6	68.4 ± 15.6	0.520
Sex ratio (male/female)	1.08	1.13	0.898
CRP (mg/L ± SD)	5.0 ± 6.9	4.7 ± 7.4	0.958
Plasma creatinine (*μ*mol/L ± SD)	76.2 ± 16.5	117.7 ± 55.8	**<0.0001**
eGFR MDRD (mL/min ± SD)	86.2 ± 24.4	62.1 ± 24.5	**<0.0001**
Bulbar onset (%)	40.8		
Body weight at diagnosis (kg ± SD)	67 ± 14		

Differences were considered significant at *P* < 0.05*.

**Table 2:** Evolution parameters in ALS patients at diagnosis and after one year of evolution.

	At diagnosis	After 1 year of evolution	Average decrease after 1 year of evolution
ALSFRS (/48) (mean ± SD)	37 ± 7	28 ± 8	10 points
Forced vital capacity (%) (mean ± SD)	82 ± 29	70 ± 29	20%

**Table 3:** Levels of biochemical compounds involved in iron metabolism according to disease status (means ± standard deviations).

Mean ± SD	ALS (*n* = 104)	Controls (*n* = 145)	*P* value	*P* for men ALS *n* = 54 controls *n* = 77	*P* for women ALS *n* = 50 controls *n* = 68
Serum iron (*μ*mol/L)	16.7 ± 5.0	14.7 ± 4.9	**0.002** ∗	**0.033** ∗	**0.013** ∗
Serum transferrin (g/L)	2.27 ± 0.4	2.32 ± 0.4	0.407	0.573	0.546
TIBC (*μ*mol/L)	56.6 ± 9.4	58.2 ± 10.0	0.370	0.485	0.543
TSC (%)	29.0 ± 9.8	25.9 ± 10.3	**0.017** ∗	0.151	**0.041** ∗
Serum ferritin (*μ*g/L)	202.2 ± 142.0	129.6 ± 83.2	**<0.0001** ∗	**0.0001** ∗	**0.041** ∗

Differences were considered significant at *P* < 0.05*.
